# Novel histone deacetylase inhibitor MPT0G009 induces cell apoptosis and synergistic anticancer activity with tumor necrosis factor-related apoptosis-inducing ligand against human hepatocellular carcinoma

**DOI:** 10.18632/oncotarget.6352

**Published:** 2015-11-07

**Authors:** Mei-Chuan Chen, Hui-Hsuan Huang, Chin-Yu Lai, Yi-Jyun Lin, Jing-Ping Liou, Mei-Jung Lai, Yu-Hsuan Li, Che-Ming Teng, Chia-Ron Yang

**Affiliations:** ^1^ Ph.D. Program for the Clinical Drug Discovery from Botanical Herbs, College of Pharmacy, Taipei Medical University, Taipei, Taiwan; ^2^ Graduate Institute of Pharmacognosy, College of Pharmacy, Taipei Medical University, Taipei, Taiwan; ^3^ Pharmacological Institute, College of Medicine, National Taiwan University, Taipei, Taiwan; ^4^ School of Pharmacy, College of Medicine, National Taiwan University, Taipei, Taiwan; ^5^ School of Pharmacy, College of Pharmacy, Taipei Medical University, Taipei, Taiwan; ^6^ Center for Translational Medicine, Taipei Medical University, Taipei, Taiwan

**Keywords:** HDAC, apoptosis, FLIP, TRAIL

## Abstract

Hepatocellular carcinoma (HCC) is a frequent cause of cancer-related death; therefore, more effective anticancer therapies for the treatment of HCC are needed. Histone deacetylase (HDAC) inhibitors serve as promising anticancer drugs because they can induce cell growth arrest and apoptosis. We previously reported that 3-[1-(4-methoxybenzenesulfonyl)-2,3-dihydro-1*H*-indol-5-yl]-N-hydroxyacrylamide (MPT0G009)—a novel 1-arylsulfonyl-5-(N-hydroxyacrylamide)indolines compound—demonstrated potent pan-HDAC inhibition and anti-inflammatory effects. In this study, we evaluated the anti-HCC activity of MPT0G009 *in vitro* and *in vivo*. Growth inhibition, apoptosis, and inhibited HDAC activity induced by MPT0G009 were more potent than a marketed HDAC inhibitor SAHA (Vorinostat). Furthermore, MPT0G009-induced apoptosis of Hep3B cells was characterized by an increase in apoptotic (sub-G1) population, loss of mitochondrial membrane potential, activation of caspase cascade, increased levels of pro-apoptotic protein (Bim), and decreased levels of anti-apoptotic proteins (Bcl-2, Bcl-x_L_, and FLICE-inhibitory protein); the downregulation FLIP by MPT0G009 is mediated through proteasome-mediated degradation and transcriptional suppression. In addition, combinations of tumor necrosis factor-related apoptosis-inducing ligand (TRAIL) with lower concentrations (0.1 μM) of MPT0G009 were synergistic in cell growth inhibition and apoptosis in HCC cells. In the *in vivo* model, MPT0G009 markedly reduced Hep3B xenograft tumor volume, inhibited HDAC activities, and induced apoptosis in the Hep3B xenografts. Our results demonstrate that MPT0G009 is a potential new candidate drug for HCC therapy.

## INTRODUCTION

Hepatocellular carcinoma (HCC) is one of the most common types of life-threatening human malignant tumor [1]. Surgical resection has been considered the optimal treatment approach; however, only a small proportion of patients benefit from surgery, and a high recurrence rate is reported. Despite the approval and clinical application of new drugs such as sorafenib for advanced HCC, the overall therapeutic outcome remains poor and the prognosis is generally unsatisfactory [2]. In particular, advanced HCC cells have been known to respond poorly to the induction of apoptosis by chemotherapeutic agents due to escaping of the cellular apoptotic pathway [3]. Thus, strategies to lower the thresholds for triggering apoptosis in HCC may result in new and more effective therapeutic regimens.

Tumor necrosis factor (TNF)-related apoptosis-inducing ligand (TRAIL), a type II transmembrane protein, belongs to the TNF family. TRAIL can bind to death receptor (DR) 4 and 5 to trigger the extrinsic pathway of apoptosis [4]. TRAIL is a promising anticancer agent because of its ability to induce apoptosis in various tumor cell types while showing only negligible effects on normal cells [5]. However, several tumor types including HCC exert a profound resistance to TRAIL treatment, majorly due to the constitutive expression of intracellular TRAIL resistance-mediating factors, such as FADD-like interleukin-1β-converting enzyme (FLICE)-inhibitory protein (FLIP), in cancer cells [6, 7]. FLIP resembles the structure of caspases 8 but lacks the functional caspase domain; therefore, FLIP interferes with TNF-α, FasL, and TRAIL-induced apoptotic pathways by binding to the Fas-associated death domain (FADD) and caspase 8, which in turn, inhibits the death-inducing signal complex (DISC) formation and subsequent activation of the caspase cascade [8, 9]. Furthermore, FLIP induces cellular functions including increased cell proliferation and tumorigenesis [10]. Therefore, several studies have focused on attenuating the inhibition of cellular apoptosis through FLIP downregulation, which increases the sensitivity to TRAIL [11, 12], suggesting its promising therapeutic potential in HCC treatment.

Histone deacetylases (HDACs) are the key enzymes of epigenetic regulation by post-translational modifications of core histone or non-histone proteins; HDACs are considered to be new and promising anticancer targets, and four HDAC inhibitors have been approved for clinical use. We previously synthesized 1-arylsulfonyl-5-(N-hydroxyacrylamide)indoline derived HDAC inhibitors. Among these compounds, 3-[1-(4-methoxybenzenesulfonyl)-2,3-dihydro-1*H*-indol-5-yl]-N-hydroxyacrylamide (MPT0G009) exhibited potent anti-arthritic effects and superior pharmacokinetic parameters, indicating its potential as a therapeutic agent [13, 14]. However, the molecular action of MPT0G009 in the inhibition of cancer growth has not been clearly elucidated. In this study, we examined the antitumor activities of MPT0G009 in human HCC cells using *in vitro* and *in vivo* models and determined whether combining MPT0G009 with TRAIL could enhance sensitivity to TRAIL in HCC cells.

## RESULTS

### MPT0G009 inhibited cell proliferation and viability in human HCC cells

**Figure 1:**
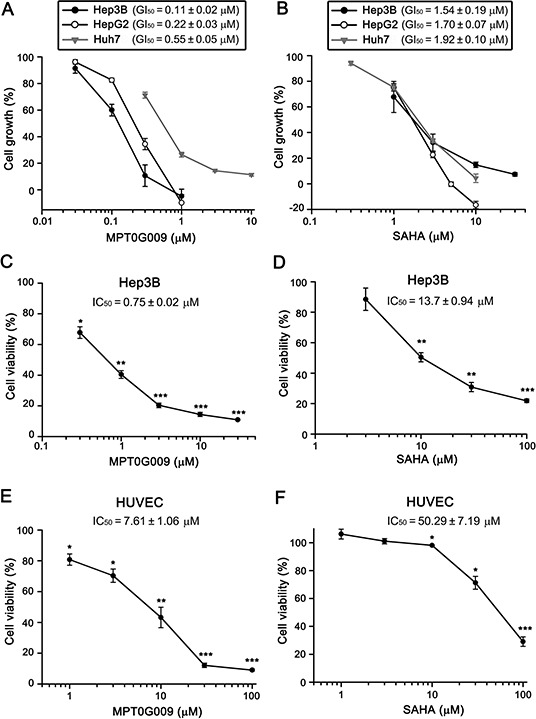
MPT0G009 inhibits cell proliferation and viability in human HCC cells **A, B.** Hep3B, HepG2, and Huh7 cells were incubated with indicated concentrations of MPT0G009 (A) or SAHA (B) for 48 h. Cell proliferation was evaluated by SRB assay. **C–F.** Hep3B cells or HUVECs were exposed to MPT0G009 (C, E) or SAHA (D, F) at the indicated concentrations for 48 h, and cell viability was measured by MTT assay. Results represent mean ± SEM. *p < 0.05, **p < 0.01, and ***p < 0.001 compared with untreated group (*n* = 3).

In a previous study, we revealed the structure of MPT0G009 and it was shown to exhibit a potent inhibitory effect on pan-HDAC enzymatic activities [14]. In this study, we first used the sulforhodamine B (SRB) assay to examine whether MPT0G009 could inhibit HCC growth. As shown in Figure 1A, in the three HCC cell lines, Hep3B, HepG2, and Huh7, MPT0G009 treatment inhibited tumor proliferation in a dose-dependent manner; the GI_50_ values (0.11 ± 0.02, 0.22 ± 0.03, and 0.55 ± 0.05 μM, respectively) were more lower than those for SAHA (1.54 ± 0.19, 1.70 ± 0.07, and 1.92 ± 0.10 μM, respectively; Figure 1B), indicating higher potency of MPT0G009. Furthermore, the cytotoxic effect of MPT0G009 was determined by MTT assay: MPT0G009 caused 50% cell death at a concentration of 0.75 ± 0.02 μM in Hep3B (Figure 1C) or 7.61 ± 1.06 μM in normal human umbilical vein endothelial cells (HUVECs; Figure 1E). In contrast, the half maximal inhibitory concentration (IC_50_) of SAHA was 13.7 ± 0.94 μM (Figure 1D) or 50.29 ± 7.19 μM (Figure 1F), respectively, in both cells. These results indicated that MPT0G009 has potential for inhibition of cell proliferation and viability in HCC cells and that it specifically targets malignant tumor cells.

### MPT0G009 inhibited HDAC activity in human Hep3B cells

**Figure 2:**
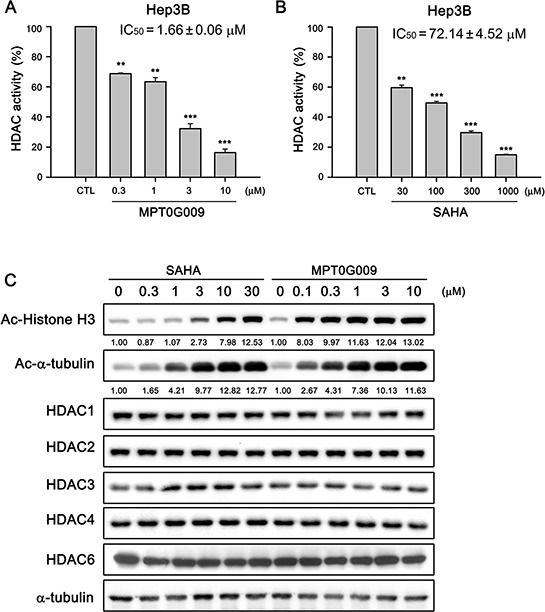
MPT0G009 inhibited HDAC activities in human Hep3B cells **A, B.** Total cell lysates of Hep3B cells were collected after treating with DMSO, indicated concentrations of MPT0G009 (A), or SAHA (B) for 24 h. Cell lysates were subjected to fluorometric HDAC activity assay kit. Data represent the mean ± SEM. **p < 0.01 and ***p < 0.001 compared with control group (*n* = 3). **C.** Hep3B cells were either treated with DMSO or were incubated with the indicated concentrations of MPT0G009 or SAHA for 24 h. Then, cells were harvested and cell lysates were prepared for western blotting analysis of the indicated proteins.

We next determined the HDAC inhibitory effect of MPT0G009 in Hep3B cells. In Figure 2A, the HDAC inhibition IC_50_ of MPT0G009 was 1.66 ± 0.06 μM in Hep3B cells, i.e., it was at least 40-fold more potent than SAHA (IC_50_ = 72.14 ± 4.52 μM; Figure 2B). Because histone H3 and α-tubulin are downstream targets of HDAC, we examined the effects of MPT0G009 on the acetylation of histone H3 and α-tubulin in HCC cells by western blotting. As shown in Figure 2C, MPT0G009 induced a significant hyperacetylation of histone H3 and α-tubulin in a concentration-dependent manner; this effect was not mediated through modulation of HDACs expression because there were no marked changes in HDAC1, 2, 3, 4, and 6 levels in response to MPT0G009 treatment. Of note, the potency of MPT0G009 on the induction of acetylated-H3 was significantly higher than SAHA, whereas as the induction of α-tubulin acetylation was less obvious than acetylated-H3. These data are consistent with our previous study showing that MPT0G009 is more potent than SAHA in inhibiting class-I HDACs than HDAC6 [14].

### MPT0G009 significantly induced Hep3B cell apoptosis

**Figure 3:**
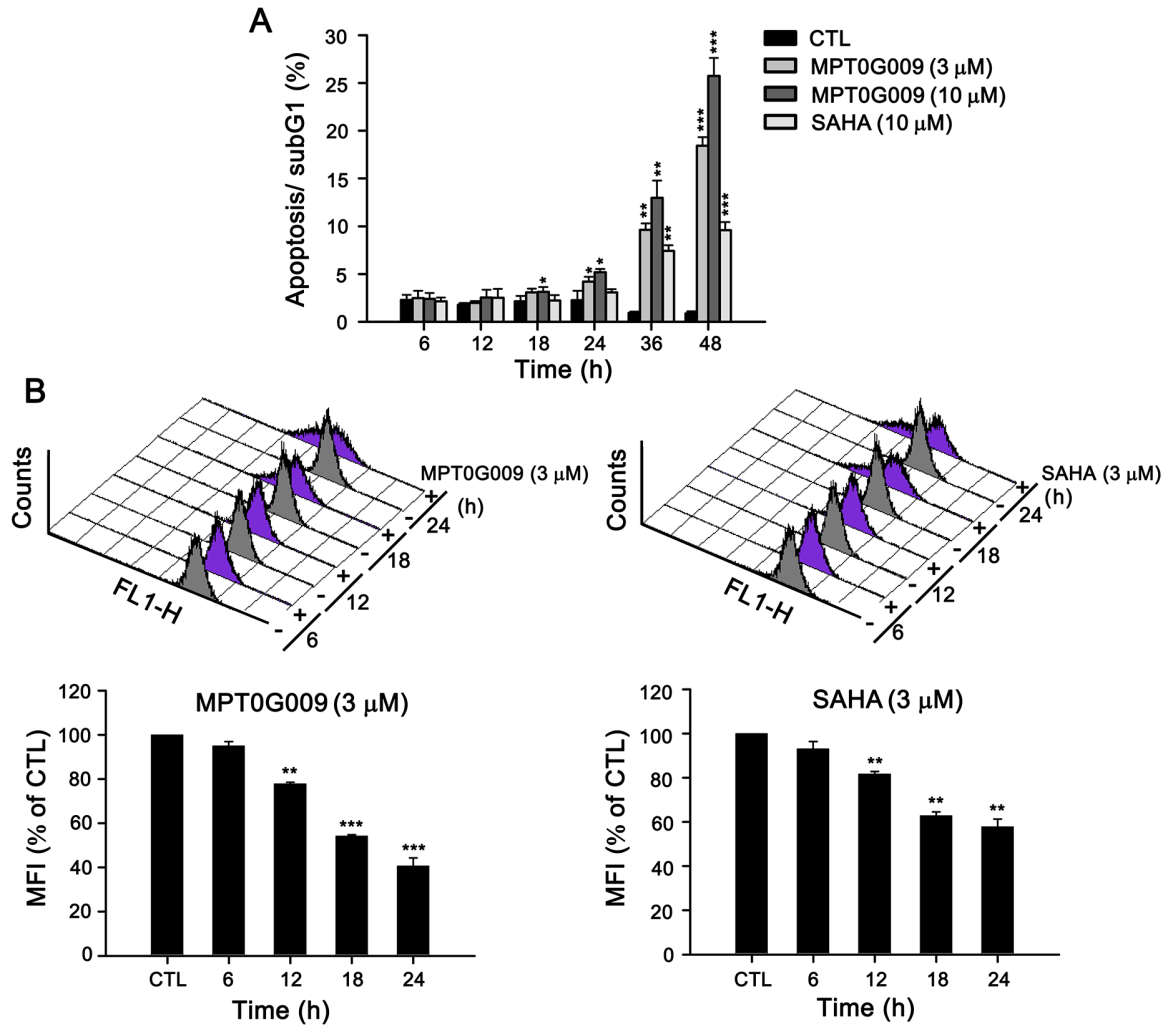
MPT0G009 induced apoptosis in Hep3B cells **A.** Hep3B cells were treated with MPT0G009 (3 or 10 μM) or SAHA (10 μM) for the indicated time, the cells were fixed, and then stained with propidium iodide to analyze the DNA contents by flow cytometry. Percentages of the sub-G1 phase in response to drug treatment are shown in (A). **B.** MPT0G009 (left panel) or SAHA (right panel; each 3 μM) triggered loss of mitochondrial membrane potential, which was determined by flow cytometric analysis with dye rhodamine 123 in Hep3B cells during different time periods. Lower panel presents statistical results. MFI, mean fluorescent intensity. Data represent the mean ± SEM. *p < 0.05, **p < 0.01, and ***p < 0.001 compared with control group (*n* = 3).

**Figure 4:**
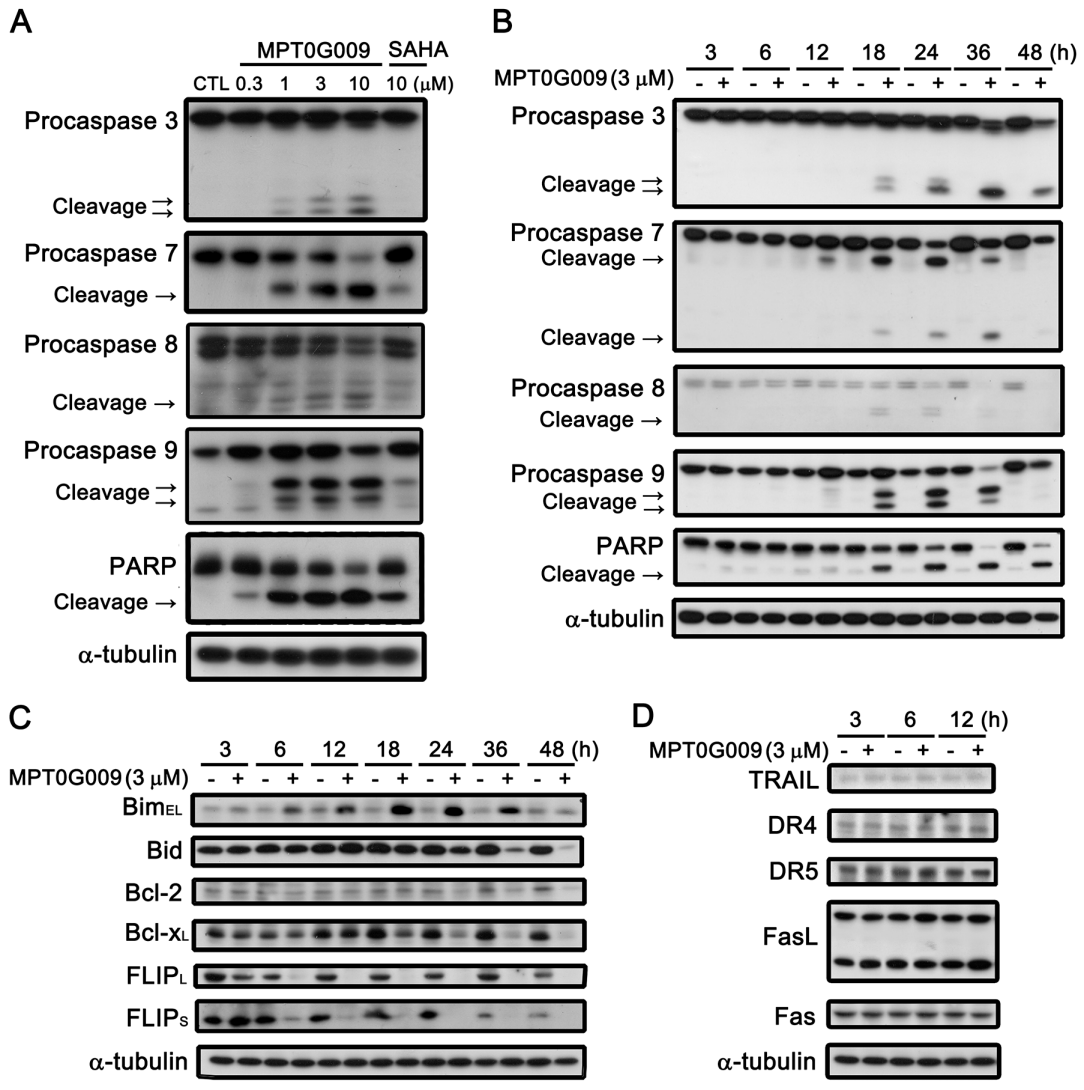
Effect of MPT0G009-induced apoptotic pathways in Hep3B cells Hep3B cells were treated with indicated agents for 24 h **A.** or MPT0G009 (3 μM) for indicated time **B.** and subjected to western blotting. **C, D.** Hep3B cells were treated with 3 μM MPT0G009 for the indicated time. Changes in pro-apoptotic proteins (Bim and Bid), anti-apoptotic proteins (Bcl-2, Bcl-x_L_, and FLIP_S/L_) (C), and death ligands (TRAIL and FasL) and death receptors (DR4, DR5, and Fas) (D) were observed.

MPT0G009 markedly inhibits tumor growth. Hence, we investigated the effect of MPT0G009 on cell cycle progression. As shown in Figure 3A and Supplemental figure 1, MPT0G009 treatment increased the number and percentage of cells in the sub-G1 phase of the cell cycle in both dose- and time-dependent manner. Of note, MPT0G009 treatment induced cell cycle arrest at G2/M phase from 24 to 48 h (Supplemental figure 2). The reference compound SAHA also produced a dose- and time-dependent increase in the percentage of cells in the sub-G1 phase, but with lesser potency compared with MPT0G009. Because mitochondrial membrane potential loss is associated with apoptosis, we determined whether MPT0G009 treatment could change mitochondrial membrane potential. Hep3B cells were exposed to 3 μM MPT0G009 for the indicated times (6, 12, 18, or 24 h) and treated with fluorescence dye rhodamine 123 for 1 h; then, the cells were analyzed by flow cytometry. As shown as Figure 3B, MPT0G009 treatment triggered significant loss of mitochondrial membrane potential starting at 12 h, which was maintained at least until after 24 h, compared with those of an untreated control; SAHA caused a similar effect, but with lesser potency than MPT0G009. In addition, MPT0G009 treatment not only increased the expression of caspase 3, 7, 8, and 9 and poly (ADP-ribose) polymerase (PARP) cleavage forms in a concentration-dependent (Figure 4A) and time-dependent (Figure 4B) manner but also increased levels of pro-apoptotic protein Bim, decreased levels of Bid proform, and downregulated expression of anti-apoptotic proteins, Bcl-2, Bcl-x_L_, and FLIP (Figure 4C). Furthermore, no changes in death ligands (TRAIL and FasL) and death receptors (DR4, DR5, and Fas) were observed in response to MPT0G009 treatment (Figure 4D) and higher TRAIL and TNF-α expressions were detected in Hep3B cells (Supplemental figure 3). Thus, MPT0G009 induced apoptosis through a caspase-dependent pathway.

### MPT0G009 synergistically enhanced TRAIL-induced cell growth inhibition and apoptosis in human HCC cells

**Figure 5:**
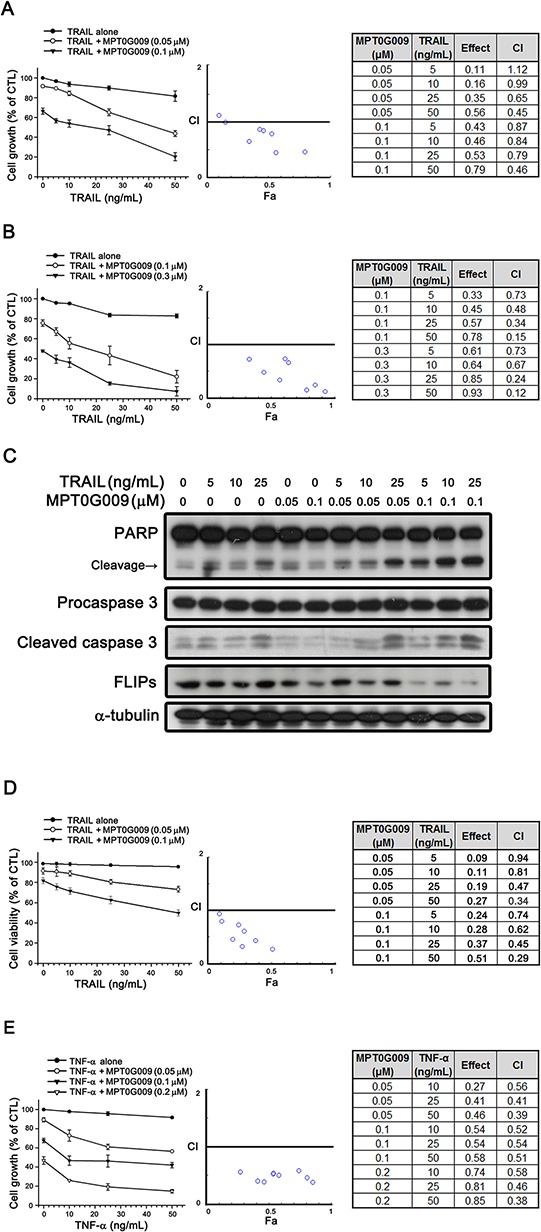
Synergistic growth-inhibition and apoptosis of MPT0G009 and TRAIL in human HCC cells **A, B.** TRAIL-resistant Hep3B (A) and Huh7 (B) cells were treated with the indicated concentrations of MPT0G009, TRAIL, or a combination of both agents for 48 h. The growth-inhibitory effect was analyzed by SRB assay (left panel). The combination index (CI) values for the combination of MPT0G009 and TRAIL were calculated by CompuSyn software (middle and right panel). **C, D.** Hep3B cells were incubated with MPT0G009 or TRAIL at indicated concentrations for 24 h, total cell lysates were subjected to western blotting for the indicated antibodies (C) or cell viability was measured by MTT assay (D) The combination index values were analyzed as (A). **E.** Hep3B cells were exposed to MPT0G009, TNF-α, or combination both agents at indicated concentrations for 48 h, and the growth inhibition and combination indices were analyzed as explained in (A). Data represent the mean ± SEM (*n* = 3).

FLIP can interrupt TRAIL, TNF-α, or FasL-induced apoptosis by binding to FADD and/or caspase 8, which in turn, prevents DISC formation and subsequent activation of the caspase cascade [9]. Our result showed MPT0G009 treatment decreased FLIP expression (Figure 4C), thus suggesting it could sensitize TRAIL-induced apoptosis. We further evaluated the apoptotic effect with a combination of MPT0G009 and TRAIL. TRAIL treatment (5, 10, 25, and 50 ng/mL) in Hep3B and Huh7 cells, which are TRAIL-resistant [15], for 48 h did not cause significant cell growth inhibition (Figure 5A and 5B); lower concentration (0.1 μM) of MPT0G009 alone caused only mild inhibition in both cells (Figure 5A and 5B). However, a significant inhibition on cell growth was observed after treatment with a combination of MPT0G009 and TRAIL compared with a single-agent group, and this combination showed a synergistic anti-proliferation effect because the combination index was evaluated by applying the Chou–Talalay method and the values were < 1.0 (Figure 5A and 5B) [16]. Furthermore, combined treatment with MPT0G009 (0.05 or 0.1 μM) and TRAIL (5, 10, and 25 ng/mL) in Hep3B cells caused marked increase in expression of cleavage form of PARP/caspase 3 and downregulation of FLIP compared with single treatment (Figure 5C), and this combination also exerts synergistic effect in apoptosis (Figure 5D, Supplemental figure 4–6). Similar synergistic cell growth inhibition was observed with an MPT0G009 and TNF-α combination in Hep3B cells (Figure 5E). Thus, MPT0G009 synergistically enhanced TRAIL-induced cell growth inhibition and apoptosis.

### MPT0G009-induced FLIP downregulation is dependent on proteasome-mediated degradation and transcriptional suppression

**Figure 6:**
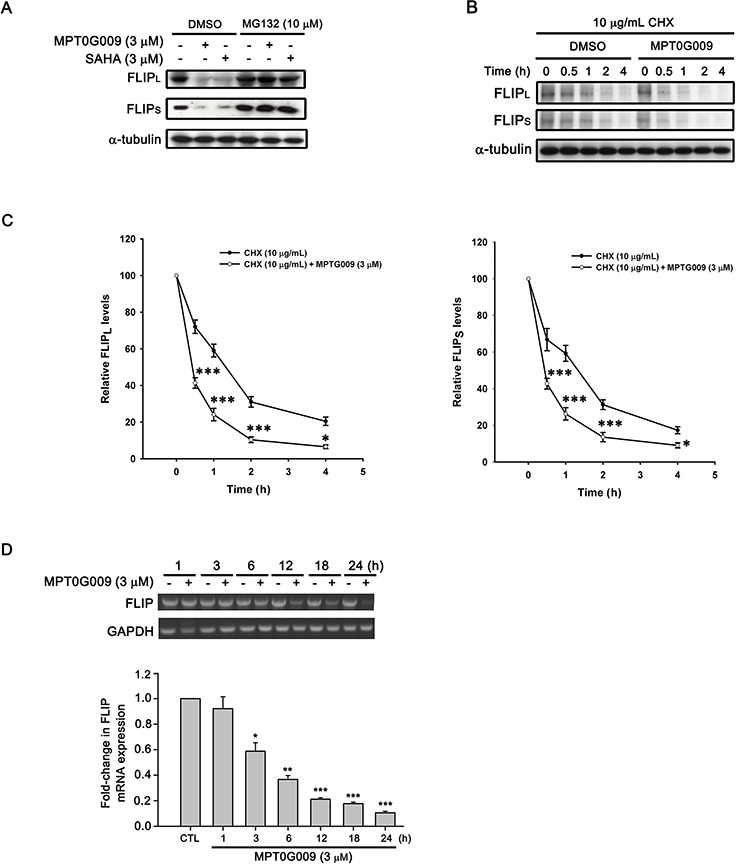
Proteasome-mediated degradation and transcriptional suppression were involved in MPT0G009-induced FLIP downregulation **A.** Hep3B cells were pretreated with 10 μM MG132 for 30 min, followed by incubation with DMSO, 3 μM MPT0G009, or 3 μM SAHA for another 6 h, and total cell extracts were subjected to western blotting. **B.** Hep3B cells were incubated with cycloheximide (CHX; 10 μg/mL) for 1 h and then combined with DMSO or MPT0G009 for indicated times. The cells were subjected to western blot analysis and the expression of FLIP_L_ and FLIP_S_ was further quantified **C.** Data represent the mean ± SEM. *p < 0.05 and ***p < 0.001 compared with the cycloheximide treatment group (*n* = 3). **D.** Cells were incubated with MPT0G009 (3 μM) for different periods as indicated, the mRNA levels of FLIP were measured by RT-PCR (upper panel) and real-time PCR (lower panel). Data represent the mean ± SEM. *p < 0.05, **p < 0.01, and ***p < 0.001 compared with the control group (*n* = 3).

**Figure 7:**
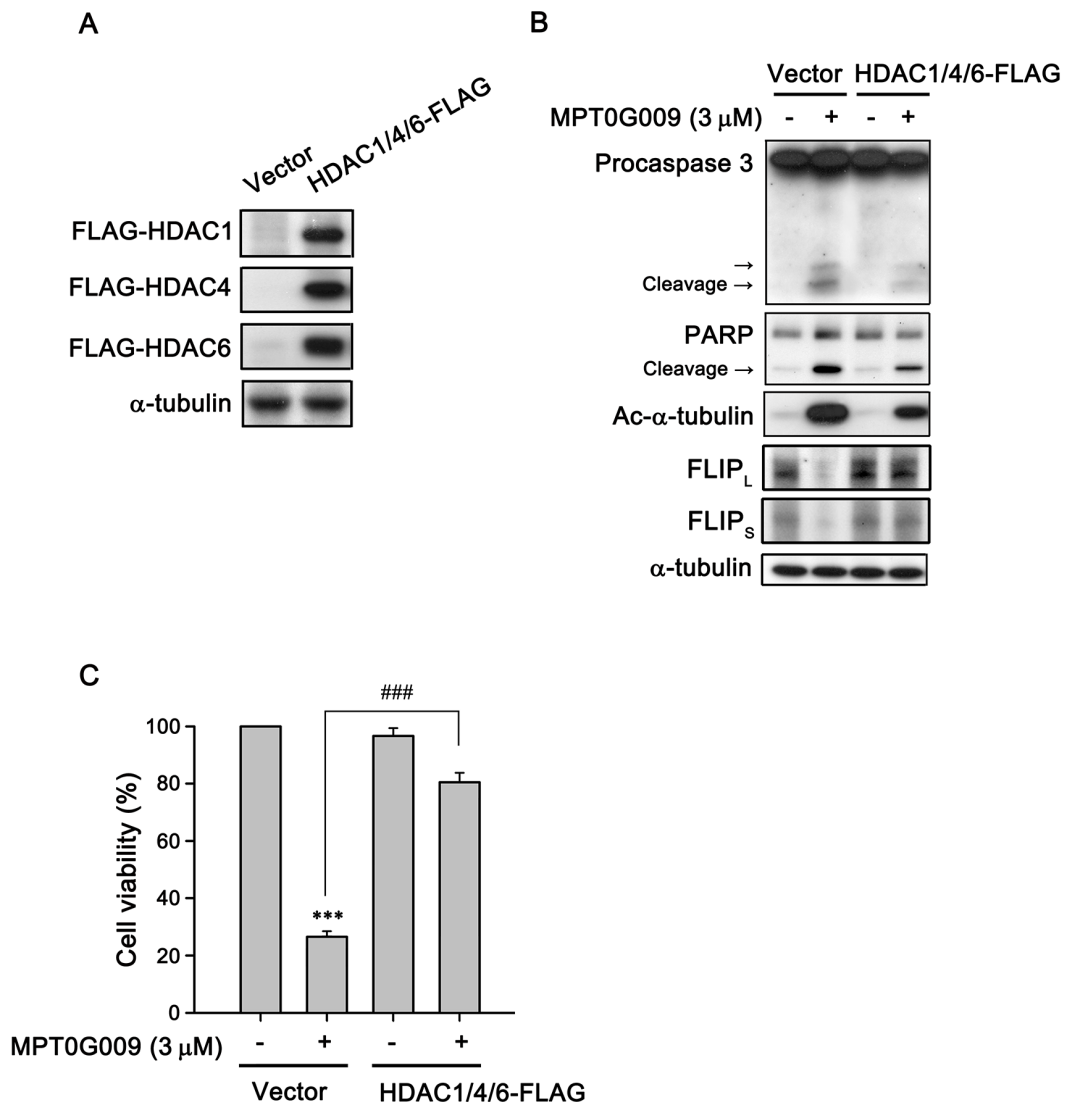
MPT0G009 induced apoptosis through HDAC inhibition **A.** Hep3B cells were co-transfected with vector or 1 μg HDAC1-, HDAC4-, and HDAC6-FLAG plasmids for 24 h; **B.** Cells transfected as in (A) before treatment with MPT0G009 (3 μM) for another 24 h. Total cell lysates were subjected to western blotting. **C.** Cells were transfected as in (A) were incubated with or without MPT0G009 (3 μM) for 48 h, then cell viability was measured by MTT assay. Results are shown as mean ± SEM. ***p < 0.001 compared with the control group; ###*p* < 0.001 compared to indicated group (*n* = 3).

FLIP degradation is reportedly correlated with the ubiquitin system [17]; therefore, we studied whether the mechanism of FLIP downregulation by MPT0G009 in HCC cells occurred through proteasome-mediated degradation. As shown as Figure 6A, MPT0G009 treatment decreased FLIP expression in Hep3B cells; however, proteasome inhibitor MG132 treatment significantly reversed MPT0G009-mediated FLIP downregulation; and no changes in cell death and NF-κB activation were observed in response to MG132 treatment (Supplemental figure 7, 8). The phenomenon was further confirmed by the examination of protein stability of FLIP in the presence of protein-synthesis inhibitor-cycloheximide. As shown as Figure 6B and 6C, FLIP proteins gradually down-regulation within 4 h after cycloheximide treatment. However, MPT0G009 treatment group significantly down-regulated the expression of FLIP while comparing with vehicle group. These results indicated that MPT0G009 reduced the protein stability of FLIP_L/S_ in Hep3B cells. Furthermore, MPT0G009 treatment also markedly decreased FLIP mRNA expression (Figure 6D). These results indicated a proteasome-degradation pathway and transcriptional suppression were involved in FLIP downregulation by MPT0G009. In addition, we examined whether MPT0G009 mediated apoptosis by inhibiting HDAC activity, we transiently transfected HDAC1-, 4-, and HDAC6-encoding plasmids to Hep3B cells, and the result indicated the expression of the expected isoforms (Figure 7A). Empty vector-transfected or HDAC1-, HDAC4-, and HDAC6-coexpressing Hep3B cells were incubated for 24 h with or without 3 μM MPT0G009; then, cell lysates were subjected to western blotting. The data showed that overexpression of HDACs significantly reduced the expression of caspasec 3, PARP cleavage and downregulation of FLIP while comparing with the MPT0G009 treatment group (Figure 7B). Moreover, ectopically expression of HDACs also showed a reversal effects on MPT0G009-induced cytotoxicity (Figure 7C). These results showed that the MPT0G009-induced apoptosis was primarily due to a decrease in HDAC activity.

### MPT0G009 inhibited growth of human HCC cells *in vivo*

**Figure 8:**
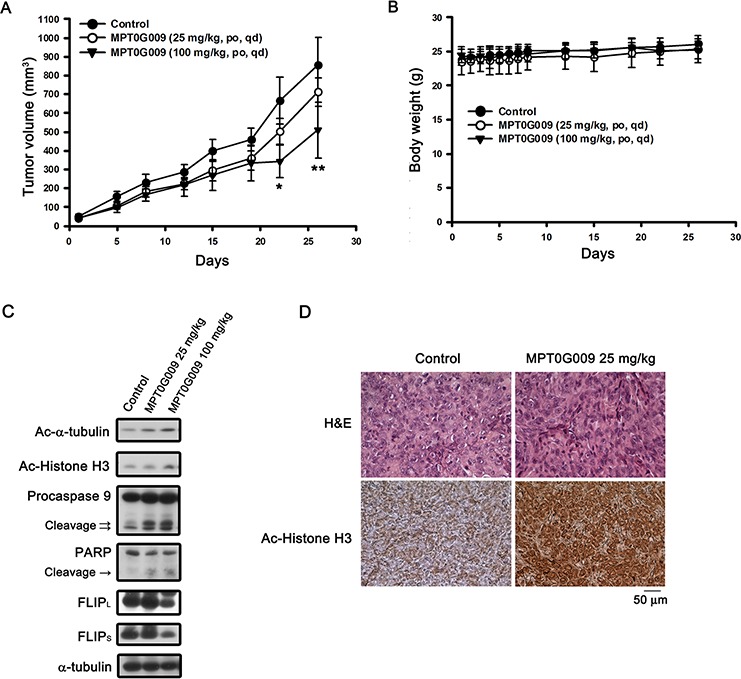
MPT0G009 suppressed human Hep3B xenografts growth *in vivo* Male nude mice bearing Hep3B tumors (~50 mm^3^) were divided into three groups and dosed as described in the Materials and Methods. The tumor volumes **A.** and body weight **B.** of mice were measured. Results are mean ± SEM. *p < 0.05, **p < 0.01 compared with control group (*n* = 8). **C.** Hep3B xenograft tumor homogenates were analyzed by western blotting. **D.** Paraffin sections of control (vehicle-treated) or MPT0G009-treated Hep3B xenografts were stained with hematoxylin and eosin or anti-acetyl-histone H3 antibody; then, sections were examined by light microscopy under × 400 magnification.

Further, we evaluated the *in vivo* inhibitory tumor growth effect of MPT0G009 using a xenograft model. Once a tumor size of 50 mm^3^ was achieved, mice were orally treated with the vehicle (control) or vehicle with MPT0G009 (25 and 100 mg/kg) once daily. As shown in Figure 8A, administration of MPT0G009 (100 mg/kg) significantly reduced tumor volume. In addition, no significant differences in weight loss were observed during MPT0G009 treatment periods (Figure 8B). Moreover, tumor homogenates prepared for western blotting showed there were significantly increased levels of acetyl-α-tubulin, acetyl-histone H3, cleavage form of caspase 9, and PARP as well as downregulation of FLIP expression in the MPT0G009 treatment group (Figure 8C). Furthermore, hyperacetylation of histone H3 was observed in the slides of the MPT0G009 treatment group (Figure 8D). These results show that MPT0G009 treatment significantly inhibited tumor growth *in vivo*.

**Figure 9:**
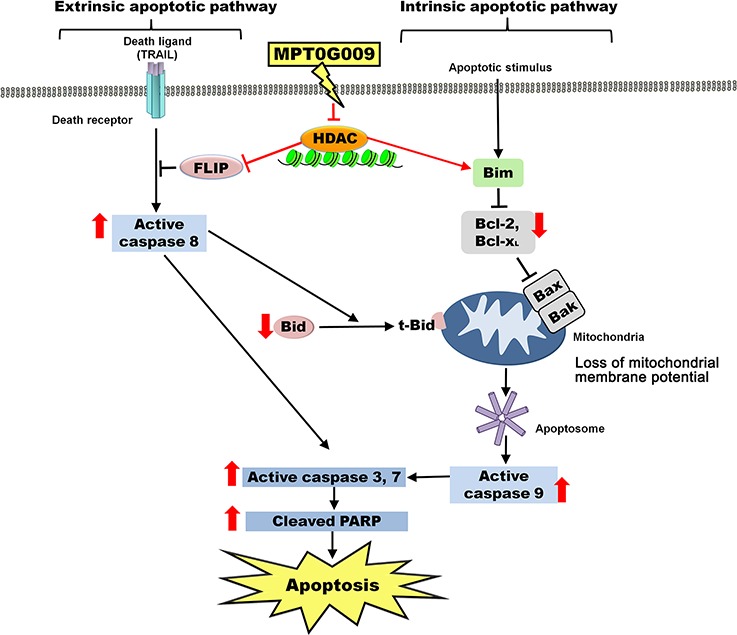
Schematic summary of MPT0G009-induced apoptosis in human HCC cells by inhibiting HDAC

Taken together, our results showed that MPT0G009 treatment significantly inhibited HCC cell growth, and induced apoptosis by activating extrinsic and intrinsic apoptotic pathways (Figure 9). Moreover, MPT0G009 treatment downregulated FLIP expression, and when combined with TRAIL, it exhibited synergistic cell growth inhibition and apoptosis. Thus, MPT0G009 has great potential as a new cancer therapeutic agent.

## DISCUSSION

Hepatocellular carcinoma is a complex and heterogeneous tumor. A characteristic property of HCC is its ability to escape apoptosis, which primarily results from a lack of response to apoptosis [3, 18] that causes HCC cells to be resistant to cell death and refractory to classical chemotherapy [19]. Therefore, new strategies to address the resistance of HCC cells to apoptosis are required. HDACs display multifaceted functions by coordinating the interaction of signal pathways with chromatin structure remodeling and the activation of non-histone proteins, which play critical roles in cellular processes including apoptosis [20]. Thus, HDAC inhibitors are considered promising agents for cancer treatment, and four HDAC inhibitors have been approved for clinical use in T-cell lymphoma and multiple myeloma therapies. In a previous study, we have evaluated the preclinical anti-arthritic effects of an orally available HDAC inhibitor, MPT0G009, both in *in vitro* and *in vivo* models. MPT0G009 was found to inhibit synoviocyte proliferation, reduce osteoclast differentiation, and ameliorate arthritis progression. In addition, MPT0G009 exhibited longer half-life and higher oral bioavailability in rats compared with SAHA [14]. In this study, our data showed that MPT0G009 treatment in three HCC cells markedly inhibited HCC cell growth and viability; both effects were more potent than SAHA. In addition, HDAC inhibitor-induced p21 (Waf1/Cip1) expression is thought to be p53 independent. Supporting data from SAHA, a pan-HDAC inhibitor, was reported to have equal effective in p53-wild type and null cells, and it does not require p53 to exert its anticancer action [21]. In the current study, we observed that MPT0G009 induces the expression of p21 and activation of caspase 9 in Hep3B (p53-null) and HepG2 cells (p53-WT; Supplemental figure 9). As shown, MPT0G009 treatment induced significant p21 expression in both cells. Moreover, MPT0G009 induced significant mitochondria membrane loss (Figure 3B) and decreased the levels of anti-apoptotic proteins (Figure 4C). These data suggests that MPT0G009 can trigger intrinsic apoptotic pathway through perturbing normal function of mitochondria.

The binding of death ligands result in the formation of death receptor signaling platforms and subsequent apoptosis initiation. Among death ligands, TRAIL has been proved to be a safe and effective biological agent for cancer therapy because TRAIL-based therapy offers a profound apoptotic effect but without causing obvious adverse effects on normal cells [5, 22]. This effect was better than that of other members of the TNF receptor ligand family, such as TNF-α and FasL, which could increase the risk of lethal systemic inflammation and hepatotoxicity [23, 24]. However, resistance to TRAIL-mediated therapy has been observed in HCC cells and indicates TRAIL treatment alone in these cells could not induce apoptosis; this defect limits its further use in clinical treatment [15]. Detailed mechanisms of resistance to TRAIL in HCC cells have not been elucidated. Recent studies have shown that FLIP is highly expressed in HCC tissues, and it can inhibit apoptotic signals by preventing the recruitment of caspase 8 at the DISC and suppress the subsequent activation of the caspase cascade [6, 8, 9, 25]. Previous results and our data (Supplemental figure 10) have shown that FLIP was constitutively expressed in Hep3B, HepG2, and Huh7 cells, which were reported to be TRAIL-resistant HCC cells [11, 26]. MPT0G009 treatment markedly downregulated FLIP levels in Hep3B cells, and this suppression was dependent on proteasome-mediated degradation and transcriptional inhibition. Previous studies have demonstrated that HDAC inhibitor mediated protein degradation by proteasome may cause by increasing hyper-acetylation of Hsp90, a chaperon protein, and further appeared to cause disruption of Hsp90-mediated folding of proteins, and lead to its subsequent degradation by proteasome [27, 28]. Furthermore, FLIP downregulation and increase Bim levels were observed after MPT0G009 treatment for 6 h, which was ahead of the appearance of the cleavage forms of caspases, and there was a decrease in the Bid proform level and in the expression of anti-apoptotic proteins Bcl-2 and Bcl-x_L_. Therefore, MPT0G009 must have modulated FLIP and Bim expression and then mediated subsequent caspase activation and decrease in anti-apoptotic proteins. In addition, MPT0G009-mediated FLIP downregulation implies that it could have potential for use with TRAIL to enhance TRAIL sensitivity in TRAIL-resistant HCC cells. The result showed that treatment with only TRAIL (5–50 ng/mL) caused mild cell growth inhibition and small quantity of cleavage forms of PARP and caspase 3 expressions. However, a combination of low-concentration (0.1 μM) MPT0G009 with TRAIL exhibited synergistic inhibition of cell growth and increase in the cleavage forms of PARP and caspase 3 levels. Moreover, this increase of caspase cascades was accompanied by marked FLIP downregulation in response to combination treatment with MPT0G009 and TRAIL. Furthermore, previous studies proposed a correlation between upregulated TRAIL death receptor DR5 expression level and restored TRAIL sensitivity by several chemotherapeutic agents in HCC cells [15] or in leukemic cell lines [29]. In our study, MPT0G009 treatment in HCC cells revealed no change of DR4, DR5, TRAIL, FasL, and Fas levels. These results suggest that MPT0G009 synergistically enhanced TRAIL-induced apoptosis by downregulation of FLIP expression, but not by alteration of the expression of death receptors or ligands. However, it still exist the possibility that MPT0G009 treatment enhance TRAIL- or TNF-α-induced cell death through other pathways (e.g. MPT0G009 induced significant mitochondria membrane loss and decreased the expressions of anti-apoptotic proteins Bcl-2, Bcl-x_L_). In addition, the results from the *in vivo* xenograft model also revealed that MPT0G009 treatment significantly inhibited growth of HCC cells without any obvious weight loss. Furthermore, marked downregulation of FLIP levels and increased cleavage forms of caspase 9/PARP were observed in tumor homogenates.

Angiogenesis is the formation of new blood vessels from the existing vasculature and is required for the progression of tumor growth and metastasis. It has been reported that the growth of HCCs depend on their ability to recruit blood vessels, and VEGF is critical in this process. Sorafenib, the anti-angiogenic therapy, was the first systemic therapy approved for patients with advanced-stage HCC [30]. Recently, HDAC inhibitors are considered as a new type of anti-cancer therapeutics, which show promising results in pre-clinical studies and early phase clinical trials. MPT0G013, a novel HDAC inhibitor, was reported by our colleague to inhibit tumor angiogenesis through up-regulation of TIMP3 expression [31]. The GI_50_ values of MPT0G013 on Hep3B cells and HUVECs are 0.36 μM and 0.14 μM, respectively [31, 32]. In the present study, we observed that MPT0G009 exhibits greater potency on cancer cells than HUVECs with the IC_50_ values of 0.75 μM and 7.61 μM, respectively. Although the IC_50_ of MPT0G009 on HUVECs was lower than that of SAHA (Figure 1E and 1F), but the concentration used to cause cancer cells death was much lower than that on HUVECs. The data suggested that MPT0G009 is a novel anticancer agent with good safety on normal cells (Supplemental figure 11).

Taken together, the results of our study indicate that MPT0G009, a novel HDAC inhibitor, significantly induced HCC cell apoptosis and inhibited tumor growth. Furthermore, we characterized the ability of a combination of MPT0G009 with TRAIL to synergistically overcome the resistance of HCC cells toward TRAIL. These results are indicative of the possible therapeutic potential of MPT0G009 in cancer treatment.

## MATERIALS AND METHODS

### Materials

MPT0G009 and SAHA were synthesized by Dr. Jing-Ping Liou to greater than 98% purity [12]. The non-conjugated primary antibodies used were against acetyl-α-tubulin, HDAC1, HDAC2, HDAC3, HDAC4, caspase 8, caspase 9, Bid, FasL, acetyl-histone H3, phospho-NF-κB p65 (Cell Signaling Technology, Danvers, MA, USA), HDAC6, PARP, Bim_EL_, Bcl-2, Bcl-x_L_, FLIP_S/L_, α-tubulin (Santa Cruz Biotechnology Corp., Santa Cruz, CA, USA), caspase 3 and NF-κB p65 (Imgenex, San Diego, CA, USA), caspase 7 (BD Biosciences, San Jose, CA, USA), TRAIL (Acris Antibodies, San Diego, CA, USA), DR4 (BioVision Inc., Milpitas, CA, USA), DR5 (Abcam, Cambridge, MA, USA), Fas (Epitomics Inc., Burlingame, CA, USA), Flag tag (Proteintech Inc., Chicago, IL, USA). The labeled secondary antibodies were horseradish peroxidase (HRP)-conjugated anti-mouse or anti-rabbit IgG antibodies (Jackson ImmunoResearch Inc., West Grove, PA, USA). The HDAC1-FLAG (plasmid 13820), HDAC4-FLAG (plasmid 13821) and HDAC6-FLAG (plasmid 13823) plasmids were obtained from Addgene Inc. (Cambridge, MA, USA). Lipofectamine 2000 transfection reagent was from Life Technologies Co., Ltd. (Grand Island, NY, USA). Unless otherwise stated, all other chemicals were from Sigma-Aldrich (St. Louis, MO, USA).

### Cell culture

Hep3B, HepG2, Huh7, HA59T and HUVECs were purchased from Bioresource Collection and Research Center (Hsinchu, Taiwan). The cells were cultured in Roswell Park Memorial Institute medium (RPMI)1640 (Hep3B), Dulbecco's Modified Eagle's medium (DMEM) (HepG2, Huh7 and HA59T) or M199 (HUVEC) respectively supplemented with 10% (Hep3B, HepG2, Huh7 and HA59T) or 20% (HUVEC) FBS (v/v) and penicillin (100 U/mL)/streptomycin (100 μg/mL)/amphotericin B (0.25 μg/mL). All cells were maintained at 37°C in a humidified atmosphere of 5% CO_2_ in air.

### Cell proliferation assay

Cells were incubated for 48 h with the indicated concentrations of MPT0G009 or SAHA, then were fixed with 10% trichloroacetic acid, stained for 30 min with SRB (0.4% in 1% acetic acid), and washed repeatedly with 1% acetic acid, then protein-bound dye was dissolved in 10 mM Tris base solution and the optical density at 510 nm measured.

### MTT assay

Cells were seeded in 96-well plates (5,000 cells/well) and incubated overnight for attachment, and were then treated with vehicle or test compound for 48 h. After various treatments, 0.5 mg/mL of MTT was added and the plates were incubated at 37°C for an additional 2 h, then the cells were pelleted and lysed in 100 μL of dimethyl sulfoxide, the absorbance at 550 nm was measured and the values of 50% inhibition concentration (IC_50_) for each compound were determined. The combination index value was determined from the fraction-affected value of each combination according to the Chou–Talalay method by using CompuSyn software (ComboSyn, Inc.), and a combination index value below 1 represents synergism [15].

### Total HDAC enzymatic activity assay

Cells were treated with either vehicle or test compounds for 24 h. Total cell lysates were collected and the total HDAC enzyme activity was analyzed using HDAC activity fluorometric assay kit (Cat No. K330–100; BioVision, Milpitax, CA, USA). A fluorescence plate reader with excitation at 355 nm and emission at 460 nm was used to quantify HDAC activity. IC_50_ was determined at the drug concentration that results in 50% reduction of total HDAC activity when compared with the control group.

### Western blotting

Cells (1 × 10^6^) were incubated for 10 min at 4°C in lysis buffer (1 mM phenylmethylsulfonyl fluoride, 1 μg/mL of leupeptin, 5 mM sodium fluoride, 1 mM sodium orthovanadate, 10 μg/mL of aprotinin, 150 mM sodium chloride, 1 mM EDTA, 1 mM EGTA, 1% Triton X-100 and 2.5 mM sodium pyrophosphate in 20 mM Tris-HCl buffer (pH 7.4)) were scraped off, incubated on ice for an additional 10 min, and centrifuged at 17,000 g for 30 min at 4°C. Total protein was determined and equal amounts of protein were separated by electrophoresed on sodium dodecyl sulfate polyacrylamide gels (SDS-PAGE) and transferred onto a nitrocellulose membrane, which was then blocked by incubation for 30 min at room temperature with 5% fat-free milk in phosphate-buffered saline (PBS). Immunoblotting was performed by overnight incubation at 4°C with primary antibodies in PBS, followed by incubation for 1 h at room temperature with HRP-conjugated secondary antibodies. Bound antibodies were measured using ECL reagent (Advansta Corp., Menlo Park, CA, USA) and exposure to photographic film.

### Flow cytometry

The mitochondrial membrane potential was monitored by rhodamine 123. Cells were treated with test compounds for the indicated time periods; thirty minutes before the termination of incubation, the rhodamine 123 solution (final concentration of 5 mM) was added to the cells and incubated for the last 30 min at 37°C. The media were removed and cells were washed once with PBS. After detachment by trypsinization, cells were resuspended in PBS and subjected to FACScan analysis. To detect cell cycle progression, cells were harvested by trypsinization, washed with PBS, then pellets were resuspended and fixed in ethanol (70%, v/v) at − 20°C overnight, and washed once with PBS. After centrifugation, the cells were incubated for 15 minutes at room temperature in 0.1 mL of phosphate-citric acid buffer (0.2 M Na_2_HPO_4_ and 0.1 M citric acid buffer, pH 7.8). Cells were stained with propidium iodide staining buffer containing Triton X-100 (0.1%, v/v), RNase A (100 μg/mL), and propidium iodide (80 μg/mL) for 30 minutes in the dark. Cell-cycle distribution was analyzed by flow cytometry with CellQuest software (Becton Dickinson).

### Transfection

The cells were transfected with lipofectamine 2000 (Life Technologies) according to the manufacturer's protocol.

### RNA isolation, RT-PCR and real-time PCR

Total RNA was isolated from cells using TRIzol reagent (Invitrogen). Single-strand cDNA for a polymerase chain reaction (PCR) template was synthesized from 5 μg of total RNA using random primers and Moloney murine leukemia virus reverse transcriptase (Promega). The following oligonucleotide primers were used for amplification: for human cFLIP (GenBank Accession No. U97074), 5′-TTGAAGATGGACAGAAAAGCTGTGGA GACC-3′ (forward) and 5′-CACACAAAGCTGTCGTAGTCTCGGTGCTC-3′ (reverse); glyceraldehyde-3-phosphate dehydrogenase (GAPDH) (GenBank Accession No. NM_002046), 5′-TGATGACATCAAGAAGGTGGTGAAG-3′ (forward) and 5′-TCCTTGGAGGCCATGTGGGCCAT-3′ (reverse). Equal amounts (1 μg) of each reverse-transcription product were PCR-amplified using *Taq* polymerase and 35 cycles of 1 min at 95°C, 1 min at 58°C, and 1 min at 72°C. The amplified cDNA was run on 1% agarose gels and visualized under UV light following staining with SYBR Safe DNA gel stain (Invitrogen).

Quantitative PCR was performed with mastermix (TaqMan One Step RT-PCR; ABI) in a total reaction volume of 20 μl per reaction, containing 10 μL of SYBR green PCR master mix (Applied Biosystems), 5 pmol of each forward and reverse primer and 2 μL of cDNA. The oligonucleotide primers used for the amplification were as follows: for human cFLIP, 5′-GTGTATGGTGTGGATCAGACTCACT-3′ (forward) and 5′-CATGAATCTCCCATGAACATCCT-3′ (reverse); GAPDH, 5′-ATTCCACCCATGGCAAATTC-3′ (forward) and 5′-TGGGATTTCCATTGATGACAAG-3′ (reverse). GAPDH was used as endogenous control to normalize differences in total RNA levels in each sample. A threshold cycle (Ct) was observed in the exponential phase of amplification, the relative mRNA expression level was determined by calculating the ΔΔCt values and the fold change was expressed as 2^−ΔΔCt^. The value of each control sample was set at 1 and was used to calculate the fold change in target genes.

### Xenograft studies

Hep3B cells were implanted subcutaneously into eight week old male nude mice. When the tumors reached the average volume of 50 mm^3^, the mice were randomly divided into three groups (*n* = 8) and then were orally treated with the vehicle (1% carboxymethyl cellulose/0.5% Tween 80, 0.2 mL/mouse) or MPT0G009 (25, 100 mg/kg) once daily. The length (L) and width (W) of the tumor were measured by caliper every 3 to 4 days, and the tumor volume was calculated as L × W^2^/2. After twenty-six days, the animals were sacrificed and the xenografts tumor homogenates were analyzed by western blotting, or were embedded in paraffin wax then the slides were stained with hematoxylin and eosin or anti-acetyl-histone H3 antibody.

### Ethics

Animal experiments were approved by the Institutional Animal Care and Use Committee of the National Taiwan University College of Medicine (IACUC number: 20090369).

### Data analysis

The data are expressed as the mean ± SEM and were analyzed using one-way ANOVA. When ANOVA showed significant differences between groups, Tukey's post hoc test was used to determine the pairs of groups showing statistically significant differences. A *p* value of less than 0.05 was considered statistically significant.

## SUPPLEMENTARY FIGURES


