# Supplementary material for: Novel histone deacetylase inhibitor MPT0G009 induces cell apoptosis and synergistic anticancer activity with tumor necrosis factor-related apoptosis-inducing ligand against human hepatocellular carcinoma
Source: Oncotarget. 2015 Nov 7;7(1):402–17. doi: 10.18632/oncotarget.6352 (PMC4808007; doi:10.18632/oncotarget.6352)
Supplement: Supplementary file 1 [file oncotarget-07-0402-s001.pdf]

SUPPLEMENTARY FIGURES

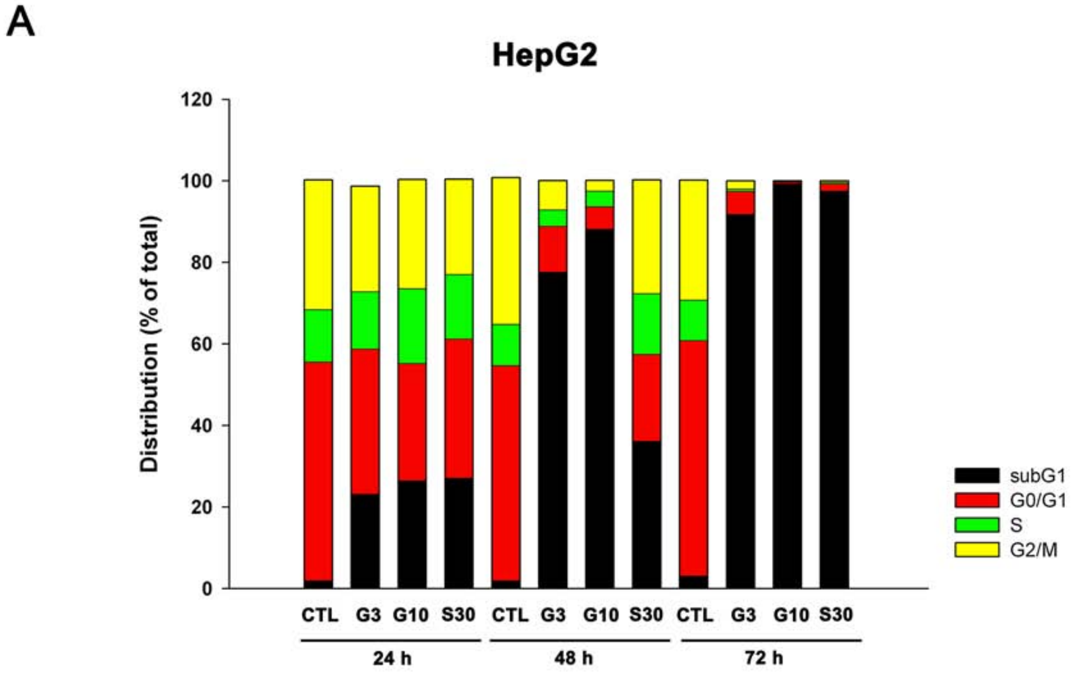

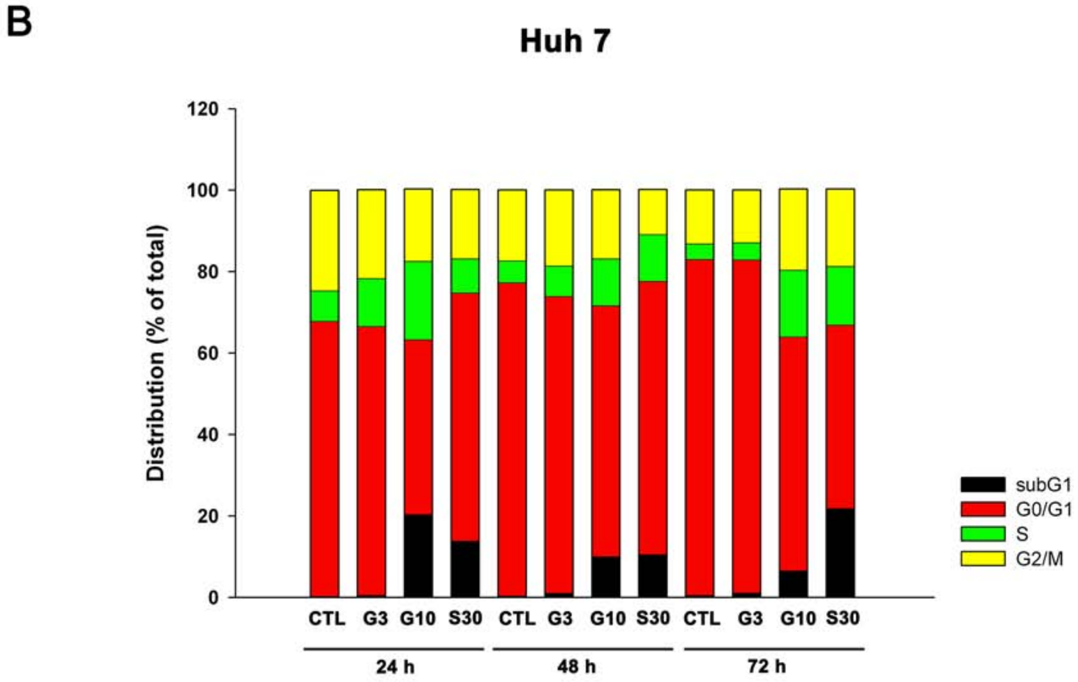

Supplementary Figure S1: The effects of MPT0G009 and SAHA on cell cycle distribution. HepG2 **A.** and Huh7 **B.** cells were treated with MPT0G009 (3 or 10 μM) or SAHA (30 μM) for the indicated time, the cells were fixed, and then stained with propidium iodide to analyze the DNA contents by flow cytometry.

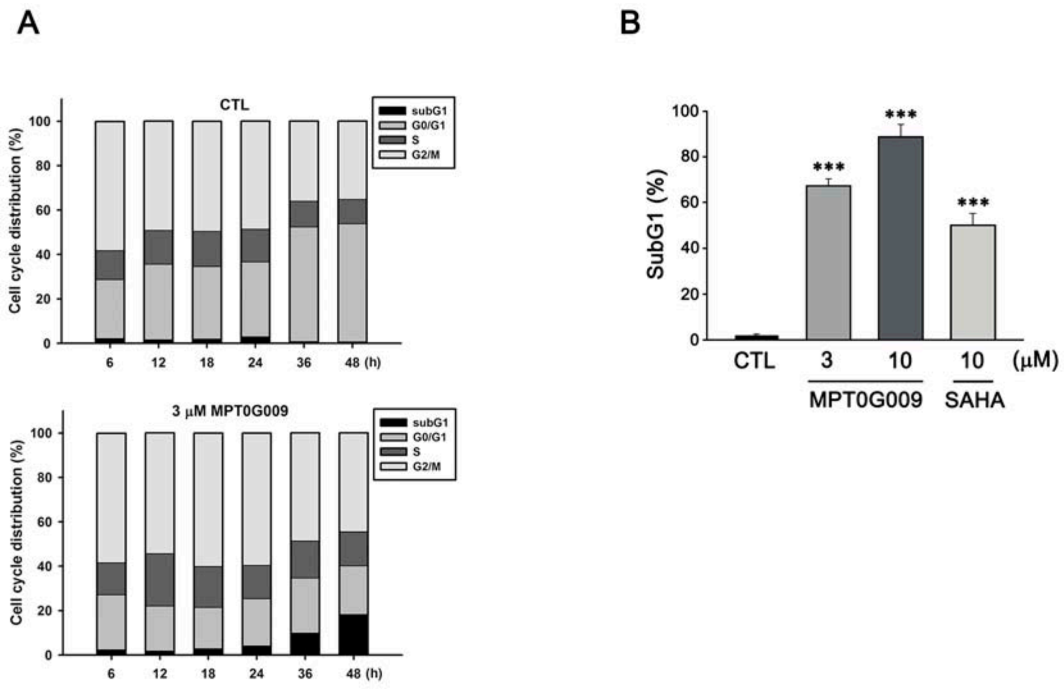

Supplementary Figure S2: MPT0G009 treatment induced apoptosis in Hep3B cells. A. Cells were incubated with or without MPT0G009 (3 μM) as indicated times, the cell cycle distributions were analyzed by flow cytometry. **B.** Percentages of subG1 phase in response to drug treated for 72 h were analyzed by flow cytometry. Data represent the mean ± SEM. ***$p < 0.001$ compared with control group ($n = 3$).

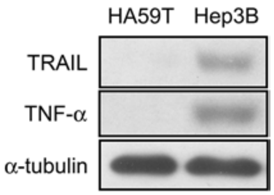

Supplementary Figure S3: Detection of TRAIL and TNF-α expression in HCC cells and normal hepatocytes. Total cell lysates of HCC Hep3B cells or normal hepatocytes HA59T cells were collected and subjected to western blot for the indicated proteins.

A

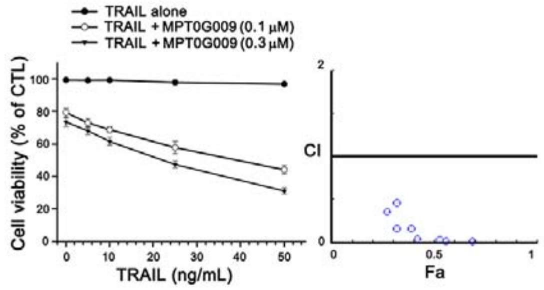

MPT0G009 (μM)	TRAIL (ng/mL)	Effect	CI
0.1	5	0.27	0.35
0.1	10	0.32	0.15
0.1	25	0.43	0.03
0.1	50	0.56	0.01
0.3	5	0.33	0.45
0.3	10	0.39	0.16
0.3	25	0.53	0.02
0.3	50	0.69	0.01

B

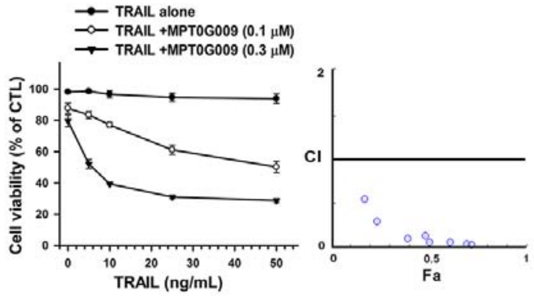

MPT0G009 (μM)	TRAIL (ng/mL)	Effect	CI
0.1	5	0.17	0.55
0.1	10	0.23	0.29
0.1	25	0.39	0.09
0.1	50	0.50	0.06
0.3	5	0.48	0.12
0.3	10	0.61	0.05
0.3	25	0.69	0.03
0.3	50	0.71	0.03

Supplementary Figure S4: Synergistic apoptosis of MPT0G009 and TRAIL in human HCC cells. Hep3B cells **A.** or HepG2 cell **B.** were incubated with MPT0G009 or TRAIL at indicated concentrations for 48 h, cell viability were measured by MTT assay. The combination index (CI) values for the combination of MPT0G009 and TRAIL were calculated by CompuSyn software (middle and right panel). Data represent the mean ± SEM (*n* = 3).

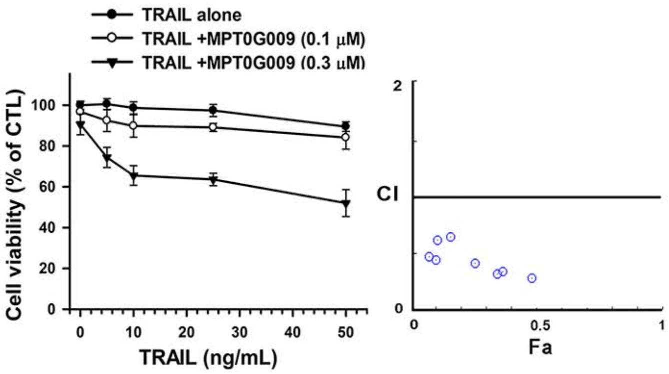

MPT0G009 (μM)	TRAIL (ng/mL)	Effect	CI
0.1	5	0.08	0.46
0.1	10	0.10	0.43
0.1	25	0.11	0.60
0.1	50	0.16	0.63
0.3	5	0.26	0.40
0.3	10	0.34	0.31
0.3	25	0.36	0.33
0.3	50	0.48	0.27

Supplementary Figure S5: Evaluation of synergistic apoptotic effect of MPT0G009 and TRAIL in human HCC cells. Hep3B cells were incubated with MPT0G009 for 6 h, and then added TRAIL at indicated concentrations for total 48 h incubation. Cell viability was evaluated by MTT assay. The combination index (CI) values for the combination of MPT0G009 and TRAIL were calculated by CompuSyn software (middle and right panel). Data represent the mean ± SEM (*n* = 3).

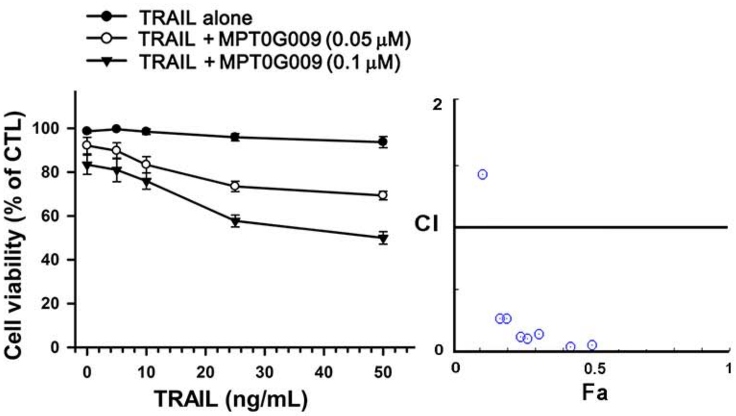

MPT0G009 (μM)	TRAIL (ng/mL)	Effect	CI
0.05	5	0.10	1.42
0.05	10	0.17	0.27
0.05	25	0.27	0.11
0.05	50	0.31	0.15
0.1	5	0.19	0.27
0.1	10	0.21	0.13
0.1	25	0.42	0.05
0.1	50	0.50	0.06

Supplementary Figure S6: Synergistic apoptosis of MPT0G009 and TRAIL in HepG2 cells. HepG2 cells were treated with MPT0G009 or TRAIL at indicated concentrations for 24 h, cell viability was measured by MTT assay. The combination index (CI) values for the combination of MPT0G009 and TRAIL were calculated by CompuSyn software. Data represent the mean ± SEM ($n = 3$).

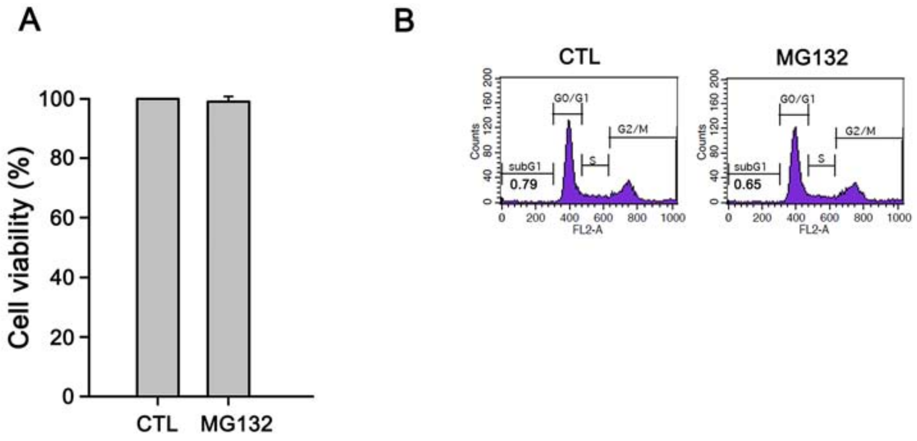

Supplementary Figure S7: The cytotoxicity evaluation of MG132 treatment in Hep3B cells. Hep3B cells were incubated with 10 μM MG132 for 6 h, and cell viability was measured by MTT assay **A.** or the cells fixed and stained with propidium iodide to analyze the DNA content by flow cytometry **B.** Results are shown as mean ± SEM ($n = 3$).

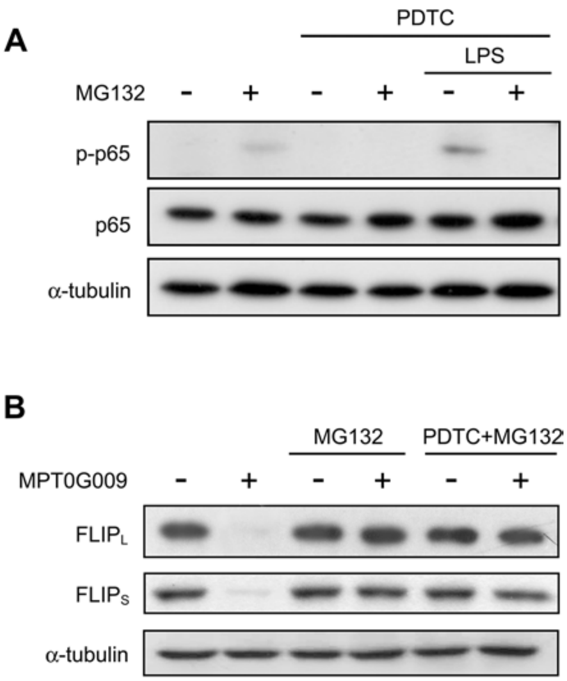

Supplementary Figure S8: Proteasome-mediated degradation was involved in MPT0G009-induced FLIP downregulation. A. Cells were incubated with pyrrolidine dithiocarbamate (PDTC; 10 µM) for 1 h, MG132 (10 µM) was added for 30 min, followed by incubation with or without 0.1 µg/mL LPS for another 1 h, and total cell extracts were subjected to western blotting. LPS was used as positive control. **B.** Hep3B cells were treated with PDTC (10 µM) for 1 h, MG132 (10 µM) was added for 30 min, followed by incubation with or without MPT0G009 (3 µM) for another 6 h; whole-cell extracts were subjected to western blot for the indicated proteins.

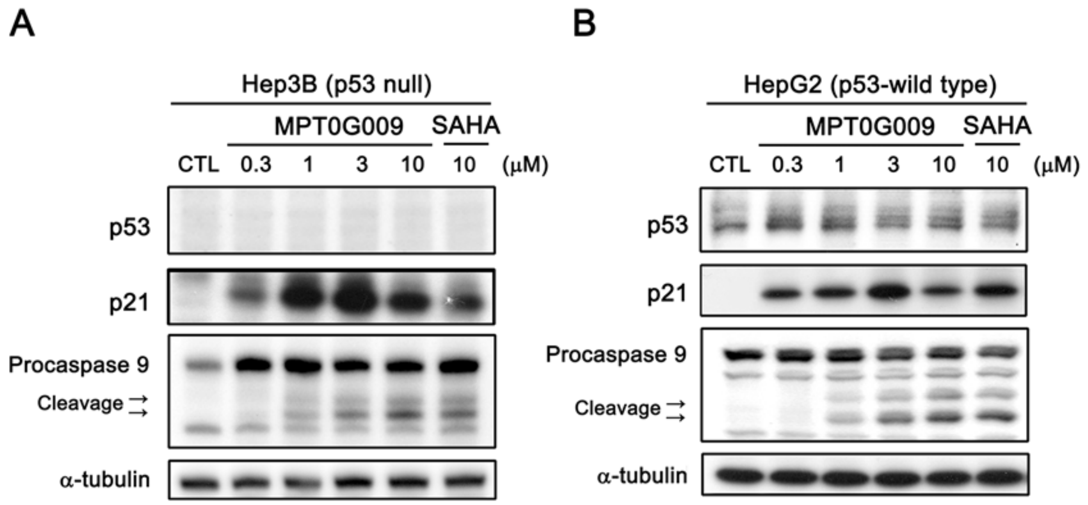

Supplementary Figure S9: Evaluation of MPT0G009 on intrinsic apoptotic pathways. Hep3B **A.** and HepG2 **B.** cells were treated with indicated agents for 24 h. Total cell lysates were prepared and subjected to western blot analysis with indicated antibodies.

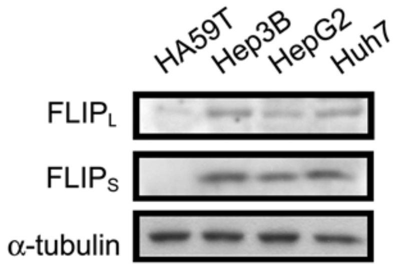

Supplementary Figure S10: FLIP expression in human normal hepatocytes and HCC cells. The FLIP expressions were observed in human normal hepatocytes HA59T cells and three HCC cell lines by western blotting.

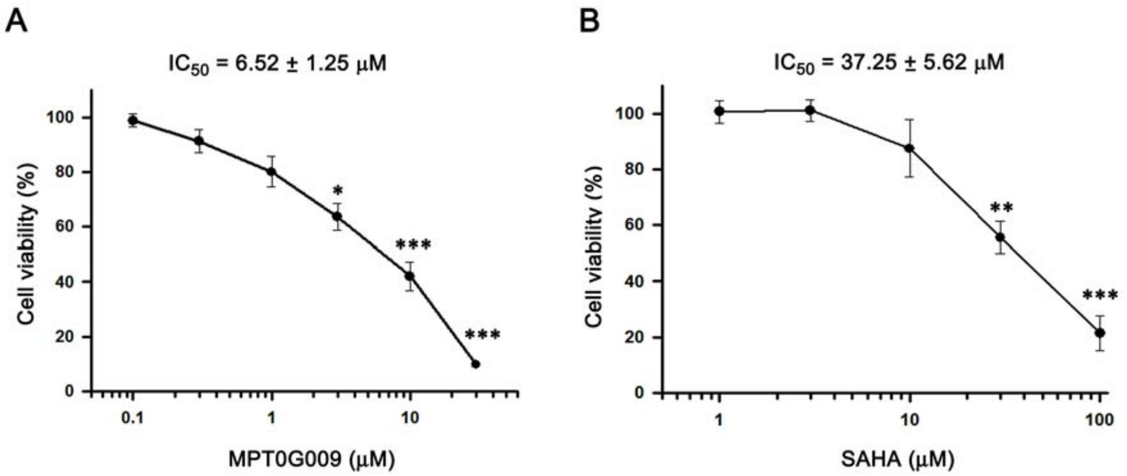

Supplementary Figure S11: Cell viability evaluation of MPT0G009 and SAHA in human hepatocytes. Human hepatocytes HA59T cells were incubated with indicated concentrations of MPT0G009 **A.** or SAHA **B.** for 48 h, the cell viability was measured by MTT assay. Results represent mean ± SEM. *$p < 0.05$, **$p < 0.01$ and ***$p < 0.001$ compared with control group ($n = 3$).
